# Validating computationally predicted TMS stimulation areas using direct electrical stimulation in patients with brain tumors near precentral regions

**DOI:** 10.1016/j.nicl.2014.03.004

**Published:** 2014-03-18

**Authors:** Alexander Opitz, Noman Zafar, Volker Bockermann, Veit Rohde, Walter Paulus

**Affiliations:** a Department of Clinical Neurophysiology, Georg-August-University, Göttingen, Germany; b Department of Neurosurgery, Georg-August-University, Göttingen, Germany

**Keywords:** Transcranial magnetic stimulation, Motor cortex, Direct electrical stimulation, Finite element method

## Abstract

The spatial extent of transcranial magnetic stimulation (TMS) is of paramount interest for all studies employing this method. It is generally assumed that the induced electric field is the crucial parameter to determine which cortical regions are excited. While it is difficult to directly measure the electric field, one usually relies on computational models to estimate the electric field distribution. Direct electrical stimulation (DES) is a local brain stimulation method generally considered the gold standard to map structure–function relationships in the brain. Its application is typically limited to patients undergoing brain surgery. In this study we compare the computationally predicted stimulation area in TMS with the DES area in six patients with tumors near precentral regions. We combine a motor evoked potential (MEP) mapping experiment for both TMS and DES with realistic individual finite element method (FEM) simulations of the electric field distribution during TMS and DES. On average, stimulation areas in TMS and DES show an overlap of up to 80%, thus validating our computational physiology approach to estimate TMS excitation volumes. Our results can help in understanding the spatial spread of TMS effects and in optimizing stimulation protocols to more specifically target certain cortical regions based on computational modeling.

## Highlights

• Use of patient specific FEM models to predict electric fields in TMS and DES.
• Validation of TMS computational models with the DES gold standard.
• TMS electric fields have high overlap with the computationally predicted DES area.
• Realistic models outperform more commonly used spherical models.
• Computational physiology approach enables a more precise preoperative mapping.

## 1 Introduction

Since its introduction (Barker et al., 1985) transcranial magnetic stimulation (TMS) became a widely used tool in cognitive and clinical neuroscience to interfere with ongoing brain activity. TMS works by applying a temporally changing magnetic field through a magnetic coil placed on the scalp, thus inducing an electric field in the brain (Barker et al., 1985; Opitz et al., 2011). This electric field acts upon neuronal structures in the brain and can lead to the initiation of action potentials that can for instance result in a motor evoked potential (MEP) when stimulating the motor cortex. The application of repetitive TMS protocols has been shown to be able to excite or inhibit a certain brain region over a time period of several minutes up to an hour and can induce long term potentiation (LTP) and long term depression (LTD) like effects (Fitzgerald et al., 2006). In cognitive studies, TMS is used either to interfere with neural circuits in a temporal precise manner which was called a “virtual lesion” (Pascual-Leone et al., 1999) or to induce longer lasting effects on neuronal activity in a specific area to study its effect on a certain behavior. For all these applications, the spatial specificity of TMS is of major importance and great efforts are employed to accurately target the intended brain area, e.g. by using a neuronavigation system. However, it is still unclear how large the stimulated area is. To what extent does the induced electric field spread to other brain regions and how can one determine the brain area that causes the functionally relevant effect? For brain areas other than the motor or visual cortex there is no direct functional output of the strength or efficacy of the stimulation and therefore, the motor cortex often serves as a brain region for testing and validating stimulation protocols. It is assumed that the findings concerning the mechanisms of action of stimulation observed at the motor cortex are valid, at least partially, for other brain areas as well. The site of stimulation of TMS was explored in many different ways by combining TMS motor mapping experiments with PET (Wassermann et al., 1996) or fMRI (Diekhoff et al., 2011; Terao et al., 1998; Weiss et al., 2012). Recently, studies were combining these methods with computational modeling of the electric field distribution using realistic finite element method (FEM) models. These models make specific predictions about the electric field distribution in the brain during TMS and might be useful in determining stimulation areas (Thielscher et al., 2011). In a recent study by Opitz et al. (2013) it was shown that computationally simulated electric fields were able to predict more than 50% of the variance of the physiological response in a motor mapping experiment. Furthermore, the combination of physiological MEP data with the modeled electric fields was able to estimate the stimulation area in brain. As the electric field is usually not restricted to a single gyrus but extends to the neighboring gyri as well (Opitz et al., 2011; Thielscher et al., 2011), it would be interesting to know which part of the stimulation area is functionally relevant or what is the threshold necessary to cause a stimulation effect.

One of the most straightforward methods to establish a structure–function relation in the brain is direct electrical stimulation (DES). Already applied nearly 150 years ago by Fritsch and Hitzig (1870) and later by Ferrier (1876) and Penfield and Boldrey (1937) it was used to establish a detailed somatotopic map of the human cortex. Today, DES is still used to investigate motor behavior, language and cognition (Desmurget et al., 2009; Desmurget et al., 2013). However, its status as a gold standard for mapping brain functions is not unchallenged because of its complex and sometimes even opposite effects at the same stimulation site (Borchers et al., 2012). In neurosurgery DES is widely used to map eloquent motor areas before tumor resection near the precentral gyrus, so that brain tissue crucial for motor control can be preserved during surgery. In many studies, DES has been compared to TMS to test if eloquent motor areas can also be reliably predicted with TMS (Krieg et al., 2012; Picht et al., 2011; Vitikainen et al., 2013) or fMRI (Forster et al., 2011) in a noninvasive manner. Similar approaches are also employed for language mapping (Sollmann et al., 2013; Tarapore et al., 2013). These studies provided valuable insights into the prediction accuracy of TMS for neurosurgical guidance (Picht et al., 2012) and established TMS as a useful tool for presurgical planning. However, the capability to precisely determine stimulated brain areas with TMS in these studies is limited as they are largely relying on either spherical models or projection based approaches to determine the stimulation area of TMS. It has been shown theoretically that these approaches are not able to capture important determining factors of TMS such as brain gyrification or coil orientation and tilts (Opitz et al., 2013; Thielscher et al., 2011).

In this study, we use individualistic high resolution finite element modeling for both TMS and DES to explore the spatial extent of the TMS effect in the brain and show how the combination of TMS with realistic FEM computational modeling can be a powerful tool to noninvasively map structure–function relationships in patients with brain pathologies.

## 2 Methods

### 2.1 Subjects

Six patients (3 men, 3 women, ages 44–79, mean 63.5 years, all right handed) with tumors in the vicinity of the motor cortex were included in the study. Five of the six patients suffered from spreading metastasis from a primary lung tumor. The other patient had an astrocytoma glioblastoma. Written informed consent was obtained before the study. All study procedures were approved by the ethics committee of the University Hospital Göttingen.

## 3 Magnetic resonance imaging

MR images were acquired at 3 T (Magnetom Trio, Siemens Medical Solutions, Erlangen, Germany) using an eight-channel head coil. Images were acquired mainly for diagnostic purposes including a T1-weighted image (MPRAGE, TR = 2000 ms, TE = 2.98 ms, flip angle = 9°, 1 × 1 × 1.1 mm resolution) and a T2-weighted image (spin echo, TR = 3200 ms, TE = 458 ms, 0.9 mm isotropic resolution). Furthermore a contrast enhanced MR image (TR = 4.6 ms, TE = 1.67 ms, flip angle = 15°, 1 mm isotropic resolution) was acquired to be used for neuronavigation during surgery.

### 3.1 TMS motor mapping

**Fig. 1:**
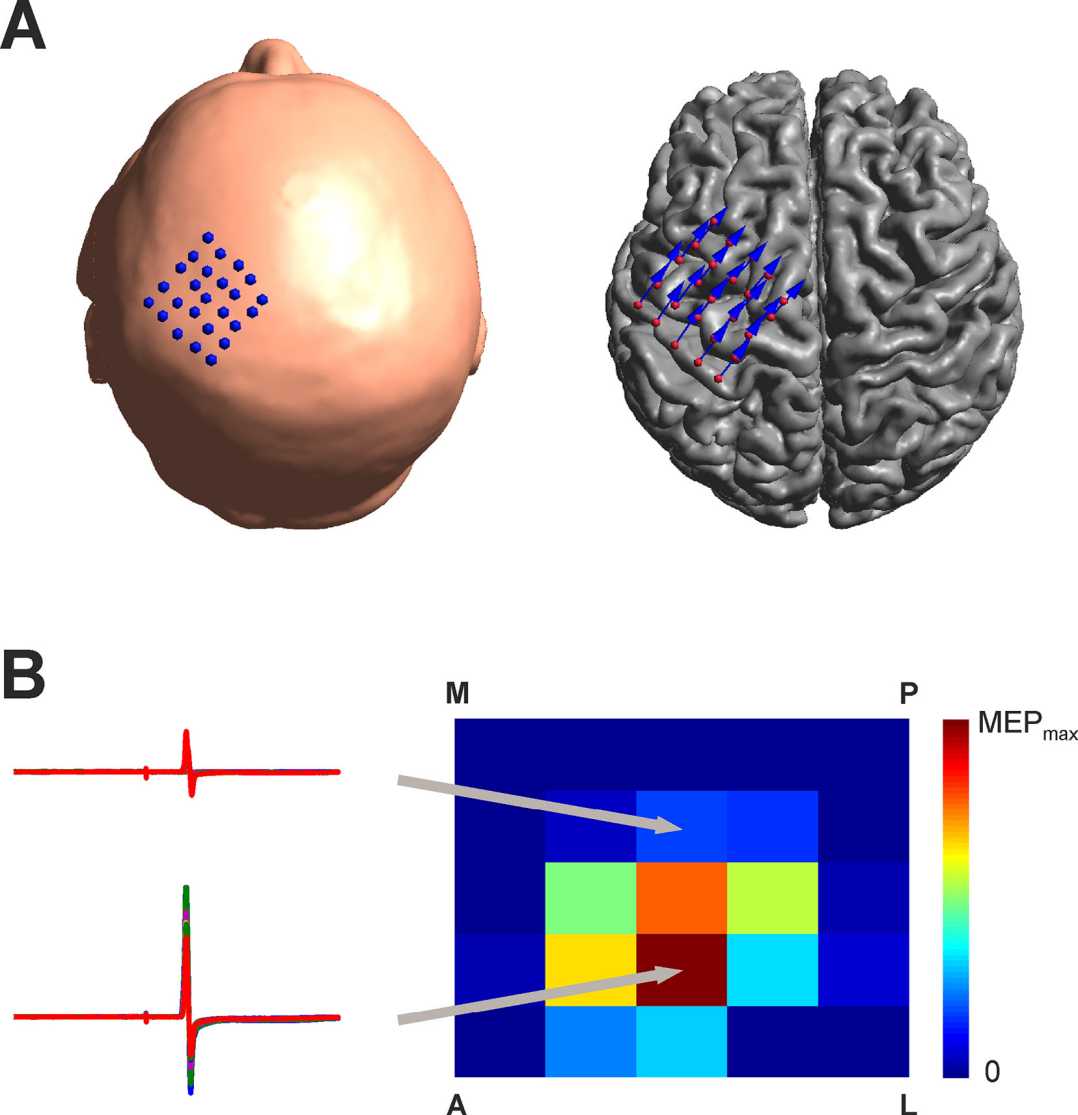
Experimental setup: A) A 5 × 5 grid (1 cm spacing) was placed on the scalp over the primary motor cortex (left panel). Orientation of the TMS coil (indicated by blue arrows) was 45° to midline for each position which is approximately perpendicular to the precentral gyrus (right panel). B) Ten motor evoked potentials (MEPs) were recorded at each position (overlaid potentials for two positions shown at the left panel). Based on the average of the MEP amplitudes a MEP map is calculated.

TMS has been conducted using a MagPro X100 stimulator with a C-B60 coil (figure-eight coil, 35 mm inner diameter, 75 mm outer diameter, 11 mm winding height, two layers of 5 windings for each wing of the coil; MagVenture, Inc., Atlanta, Georgia USA). A neuronavigation system (Visor2, ANT, Netherlands) was used to constantly monitor coil position and orientation with respect to the patient's head. Patients were seated comfortably in a reclined chair with head and arm rests. The motor hotspot was determined as the point which consistently resulted in the largest MEPs by moving the coil over the scalp. A 5 cm × 5 cm rectangular grid (1 cm spacing) centered on the initially determined hotspot was created using custom Matlab scripts (Fig. 1A left panel). Single pulse TMS with 120% resting motor threshold (RMT) of the first dorsal interosseous (FDI) hand muscle was applied at each grid point. The RMT was defined as the intensity that elicited at least 5 of 10 MEPs of at least 50 µV amplitude. In total 10 pulses with an interpulse interval of 4 s with 400 ms jitter were applied at each grid point. The coil angle applied during the whole experiment was approximately 45° to midline and recorded with the neuronavigation system (Fig. 1A right panel).

MEPs were recorded using Ag/AgCl bipolar surface electrodes placed over the FDI in a belly-tendon montage. Signals were sampled at 5 kHz and band-pass filtered between 2 Hz and 2 kHz (Fig. 1B left panel). Analog to Digital conversion was performed with a micro 1401 AD converter (Cambridge Electronic Design, Cambridge, UK). Signals were viewed with Signal 3 (Cambridge Electronic Design, v. 2.13) and stored on a computer for later offline analysis. MEP peak to peak amplitudes were averaged over each grid position and MEP maps were computed (Fig. 1B right panel).

### 3.2 Intraoperative direct electrical stimulation

**Fig. 2:**
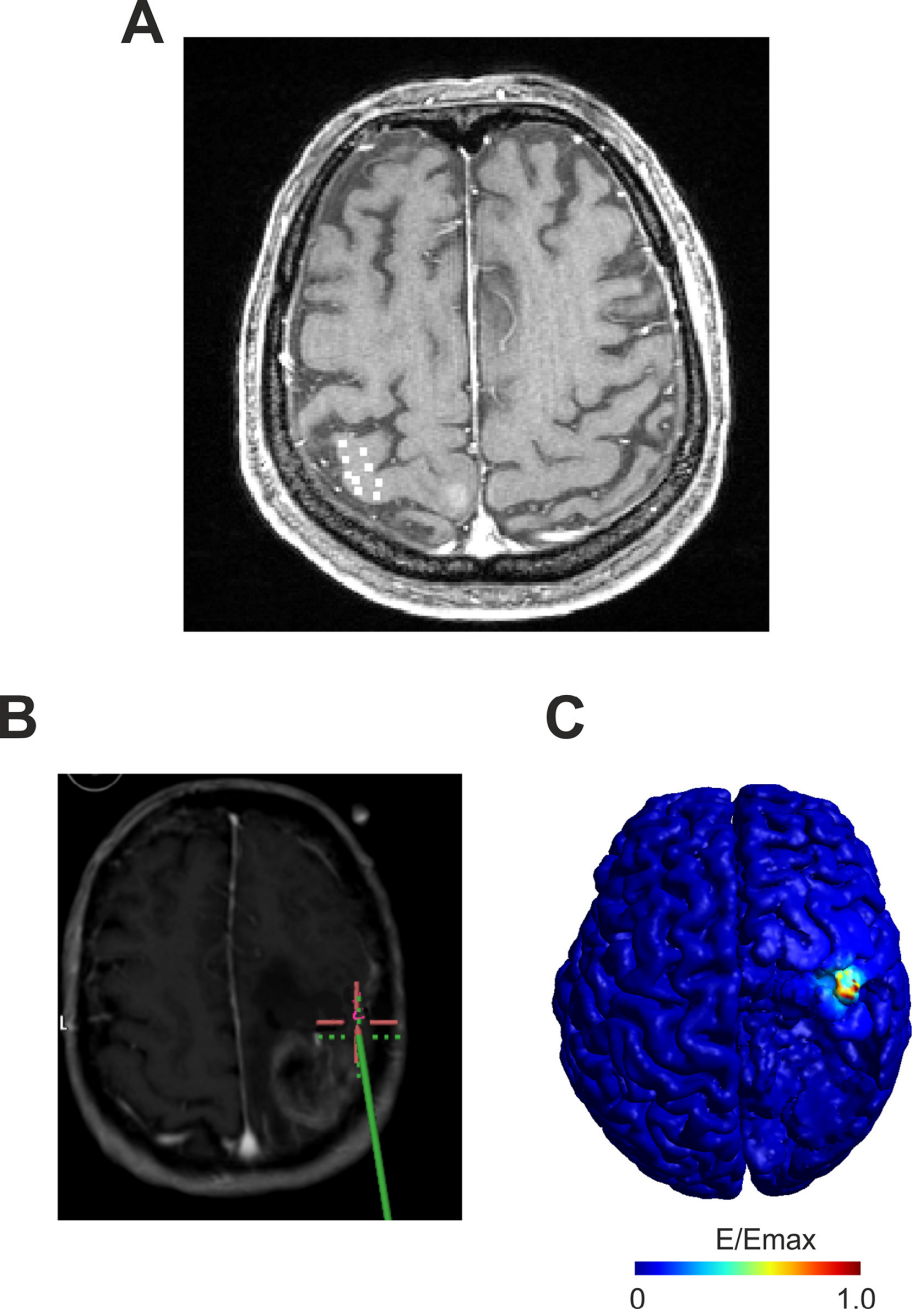
Direct electrical stimulation: A) Shown are the DES stimulation points (white squares enhanced in size for better visibility) in one example subject. B) Illustration of the intraoperative stimulation procedure. The position of the stimulation electrode is controlled by a neuronavigation software. The red cross indicates the target point at which the stimulation electrode was aimed (green cross). Different points on the motor cortex were stimulated and the elicited MEP recorded. C) Simulated electric field distribution for the DES for one stimulation point. High electric field strengths are restricted to a confined radius around the stimulation electrode.

For each patient a standard neuronavigated craniotomy was performed to get access to tumor regions. Intraoperative neuromonitoring was performed before and during operation using an Endeavor CR neuromonitoring unit (Viasys, Nicolet Biomedical, Dublin/Ohio, USA) with a monopolar brain-stimulation electrode (1.3 mm diameter, Inomed, Germany). Nine points on the surface of the precentral gyrus (with ca. 5 mm spacing, see Fig. 2A) spanning a cortical surface area of 2–4 cm^2^ were stimulated with a single anodal square pulse (pulse duration 0.2 ms). In two patients only five points could be stimulated due to constraints during the surgery. However for both patients, enough MEPs were elicited which showed a spatial variation over the stimulation points. For an illustration of the neuronavigated direct electrical stimulation procedure see Fig. 2B. Current intensities of 5 mA, 10 mA and maximally 20 mA if no response was achieved with lower intensities were applied. In addition, responses from two reference points which were located ca. 2 cm away from the other points were recorded. MEPs of the FDI hand muscle that were elicited by DES were recorded and stored offline for further analysis.

### 3.3 Computational modeling

#### 3.3.1 Realistic FEM model

**Fig. 3:**
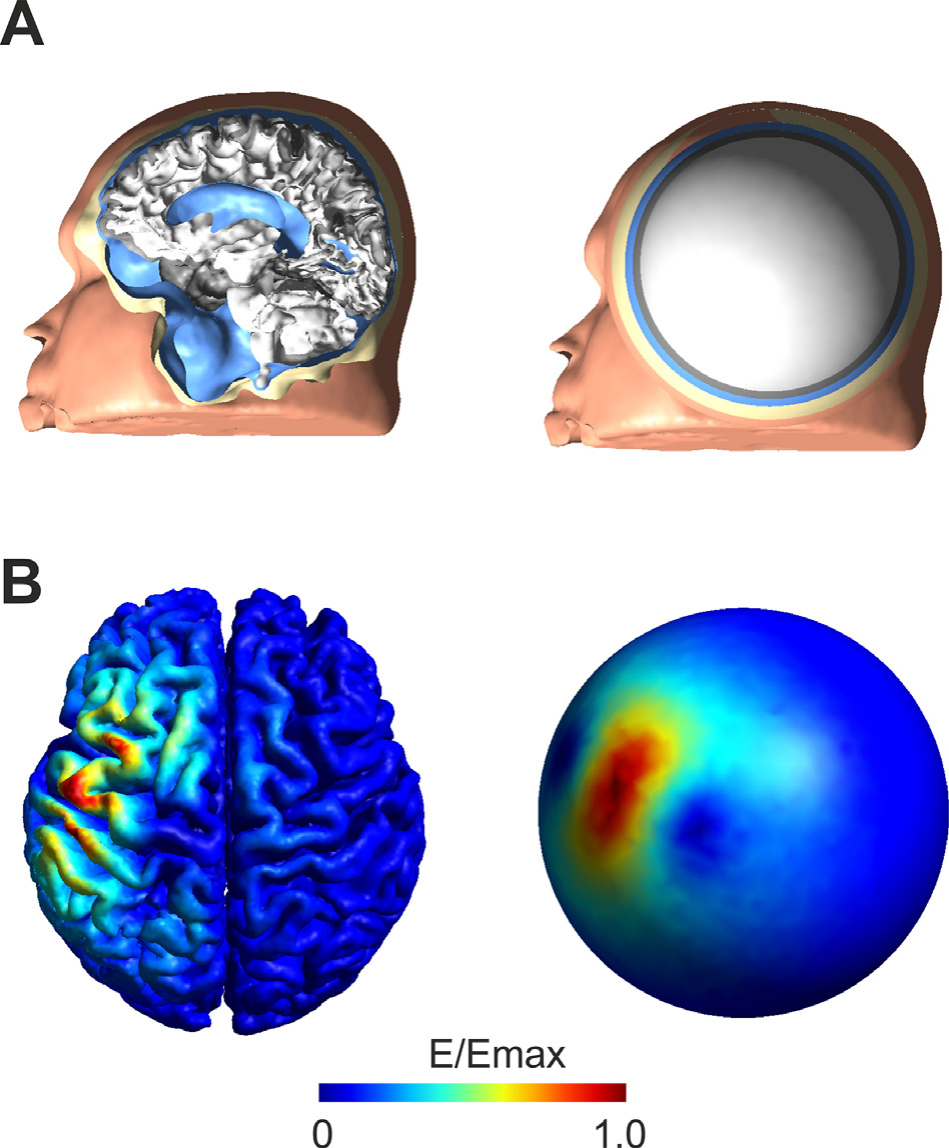
TMS computational models: A) sagittal cut through the head models for both the realistic (left panel) and the spherical (right panel) case. The surfaces of the five different tissue types are shown. The spherical model was fitted to the upper half of the skin surface of the realistic model. B) Exemplary electric field distribution in one patient for one coil position for both the realistic (left panel) and the spherical (right panel) model. While in the realistic model clear effects of tissue boundaries are visible, the electric field distribution of the spherical model is mainly determined by the primary electric field of the TMS coil.

For each patient an individual FEM model (Fig. 3A left panel) based on the T1- and T2- images was constructed using SimNibs (Windhoff et al., 2013). Failures in the automated FEM creation including missegmentations and failures in the meshing process were corrected manually when necessary. Tumor tissue in regions near the motor cortex was segmented from the MR images by intensity thresholding and corrected manually. Electric field simulations were performed in SimNibs for each coil position recorded during the TMS neuronavigated experiment (for an example see Fig. 3B left panel). Isotropic conductivities were used as follows: *σ*_skin_ = 0.465 S/m, *σ*_skull_ = 0.010 S/m, *σ*_CSF_ = 1.654 S/m, *σ*_GM_ = 0.276 S/m, and *σ*_WM_ = 0.126 S/m. Tumor conductivity was set to the conductivity of the surrounding WM tissue. We chose this value as most tumors consisted of lung tissue which has nearly the same conductivity as WM (Gabriel et al., 1996) and edemas around the tumor which might have higher conductivities were less pronounced among the patients. However, as a precise conductivity value for the tumors is hard to determine, we investigated the effect of varying tumor conductivities in more detail in one subject (see Supplementary material). Based on the simulation results, a MEP weighted mean electric field (*E_CoG_realistic_*) distribution was computed as described in Opitz et al. (2013). The rationale behind this method is that those electric field distributions that resulted in strong MEPs were stimulating functionally important brain areas while those which resulted in weak MEPs were only weakly stimulating functionally relevant brain regions.

#### 3.3.2 Spherical model

The prediction accuracy of the realistic FEM model was compared with that of a spherical model (Fig. 3A right panel) for each subject. To that end, a 5 layer spherical model as described in Thielscher et al. (2011) was fitted to the upper half of the skin surface using an ordinary least squares method (Nummenmaa et al., 2013). The upper skin surface was chosen for fitting, as it best resembles a spherical surface compared to the other tissue types. The radii (mean ± SD in mm) of the different tissues were *r*_skin_ = 88.8 ± 3.9, *r*_skull_ = 84.8 ± 4.1, *r*_csf_ = 77.8 ± 4.4, *r*_gm_ = 74.0 ± 4.8, r_wm_ = 69.8 ± 5.0. Based on the spherical model, electric field distributions were computed for all coil positions (see Fig. 3B right panel for an example). Also, an MEP weighted mean electric field distribution (*E_CoG_sphere_*) was computed for the spherical model. To compare the results between the spherical and the realistic model the values at the nodes of the spherical model in the GM and WM volume were interpolated to the realistic GM surface using a nearest neighbor approach.

#### 3.3.3 DES simulation

In addition, the electric field during DES was simulated using the realistic head models. The monopolar electrical stimulation was modeled by applying a Dirichlet boundary condition (Joucla and Yvert, 2012) for the electric potential at the stimulation point at the GM surface and a remote large return electrode at the inferior end of the FEM model. An example of the DES electric field is shown in Fig. 2C. The average electric field weighted by the MEPs recorded during surgery (*E_CoG_DES_*) was computed the same way as described for the TMS induced electric field.

#### 3.3.4 TMS–DES comparison

**Fig. 4:**
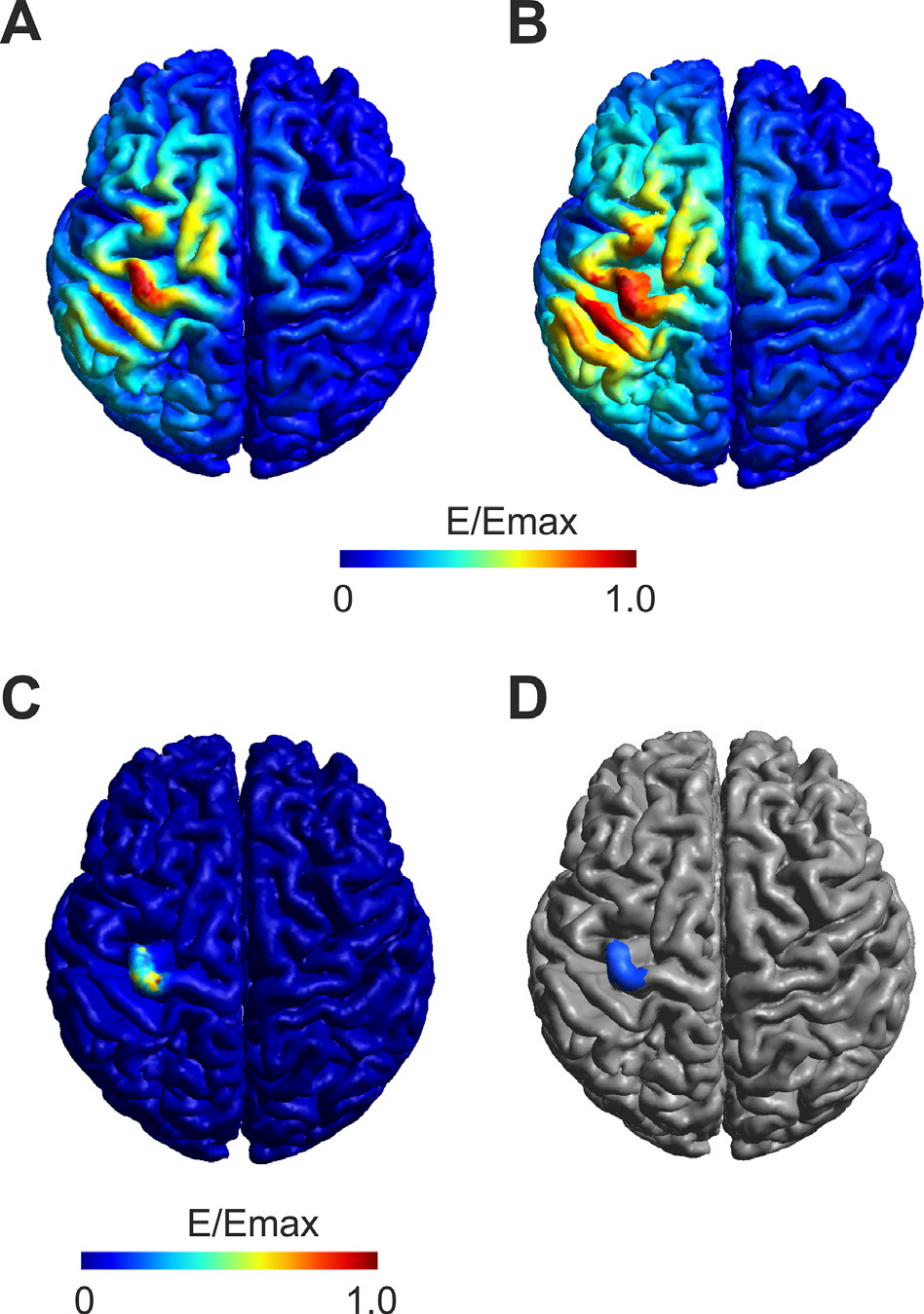
Computational predicted stimulation areas: Shown is the MEP weighted mean electric field for the A) TMS realistic model, B) TMS spherical model interpolated on the realistic GM surface and C) DES. The stimulation area in the realistic model is restricted to the crowns of the precentral gyrus as well as neighboring gyri. For the spherical model stimulation area is more extended. The stimulation area of the DES is mostly restricted to the primary motor cortex. D) Region of interest (blue area) based on the DES stimulation area (MEP weighted mean electric field > 30% of its maximum field strength).

To compare the extent of the simulated TMS electric field stimulation area that coincides with the DES stimulation area we computed the percentage of the area on the GM surface of the *E_CoG_realistic_* included in the area of the *E_CoG_DES_*. First, we determined a DES ROI by thresholding the *E_CoG_DES_* at 30% of its maximum (for an illustration of the DES ROI see Fig. 4D). This threshold was chosen as the *E_CoG_DES_* drops off fairly steep and with this chosen threshold a good coverage of the handknob region of the motor cortex was achieved. However, this choice of threshold is arbitrary and therefore we performed the same calculation with different thresholds ranging from 10% to 90% (see Supplementary material) to check the robustness of this method. In a second step, we determined the area that was covered by the *E_CoG_realistic_* exceeding a certain threshold of its maximum. We computed this area in 10% steps from 10% to 90% of the electric field maximum and determined the overlap with the DES ROI. Based on the overlap area we determined the percentage of the thresholded *E_CoG_realistic_* included in the DES ROI. The same analysis was performed with the *E_CoG_sphere_* to compare the prediction accuracy of both models. The rationale for this method is that the higher the *E_CoG_realistic_* is, the more likely it should be functionally effective and stimulate a brain area that has a causal role for generating MEPs. With higher electric field strength threshold, this area should be located in the DES ROI which serves as a gold standard for determining causal relevant areas for generating MEPs.

In a second analysis step we computed the center of gravities of the *E_CoG_realistic_* map, the *E_CoG_sphere_* map and the *E_CoG_DES_* map (in its 30% threshold ROI). This method reduces the electric field maps to one single point. In the following the Euclidian distance between these CoG points was computed. As the determination of these points also depends on the chosen electric field threshold, we again computed the distances in 10% steps from 10% to 90% of the TMS electric field maximum.

Finally, in order to acquire a simple model free estimate of the DES CoG, we made the zero order approximation that DES causes a point like excitation at the tip of the stimulation electrode. Based on this method we recomputed the DES center of gravity and the Euclidian distances to the TMS center of gravities for the realistic and spherical model.

All the described computations were performed for each subject individually and mean and standard error of mean were calculated over all subjects subsequently.

## 4 Results

Both the *E_CoG_realistic_* and the interpolated *E_CoG_sphere_* show high electric field strength in M1 (Fig. 4A and B for one example patient). For the realistic model, high electric field strengths were restricted to gyral crowns and were rapidly decreasing with increasing sulcal depth. The stimulation area was restricted to a confined region for the realistic model while for the spherical model a larger area exhibited high electric field strength. The larger stimulation area for the interpolated spherical model was independent from the applied threshold (see Table1). The *E_CoG_DES_* was considerably more spatially restricted (Fig. 4C for one example patient) towards the gyral crown of M1.

**Table 1:** Covered cortical surface area (mean ± standard error of mean over six patients) in cm^2^ depending on the threshold of the *E_CoG_* measured on the realistic model.

Electric field threshold in percent of the maximum	Spherical model	Realistic model
10%	(9.68 ± 0.29) × 10^2^	(4.67 ± 0.41) × 10^2^
20%	(3.74 ± 0.16) × 10^2^	(1.57 ± 0.18) × 10^2^
30%	(1.69 ± 0.11) × 10^2^	(7.15 ± 0.85) × 10^1^
40%	(9.35 ± 0.52) × 10^1^	(3.74 ± 0.49) × 10^1^
50%	(5.38 ± 0.30) × 10^1^	(1.94 ± 0.27) × 10^1^
60%	(3.22 ± 0.19) × 10^1^	(1.01 ± 0.13) × 10^1^
70%	(1.77 ± 0.10) × 10^1^	(4.85 ± 0.64) × 10^0^
80%	(7.07 ± 0.89) × 10^0^	(1.81 ± 0.33) × 10^0^
90%	(1.98 ± 0.38) × 10^0^	(0.59 ± 0.15) × 10^0^

**Fig. 5:**
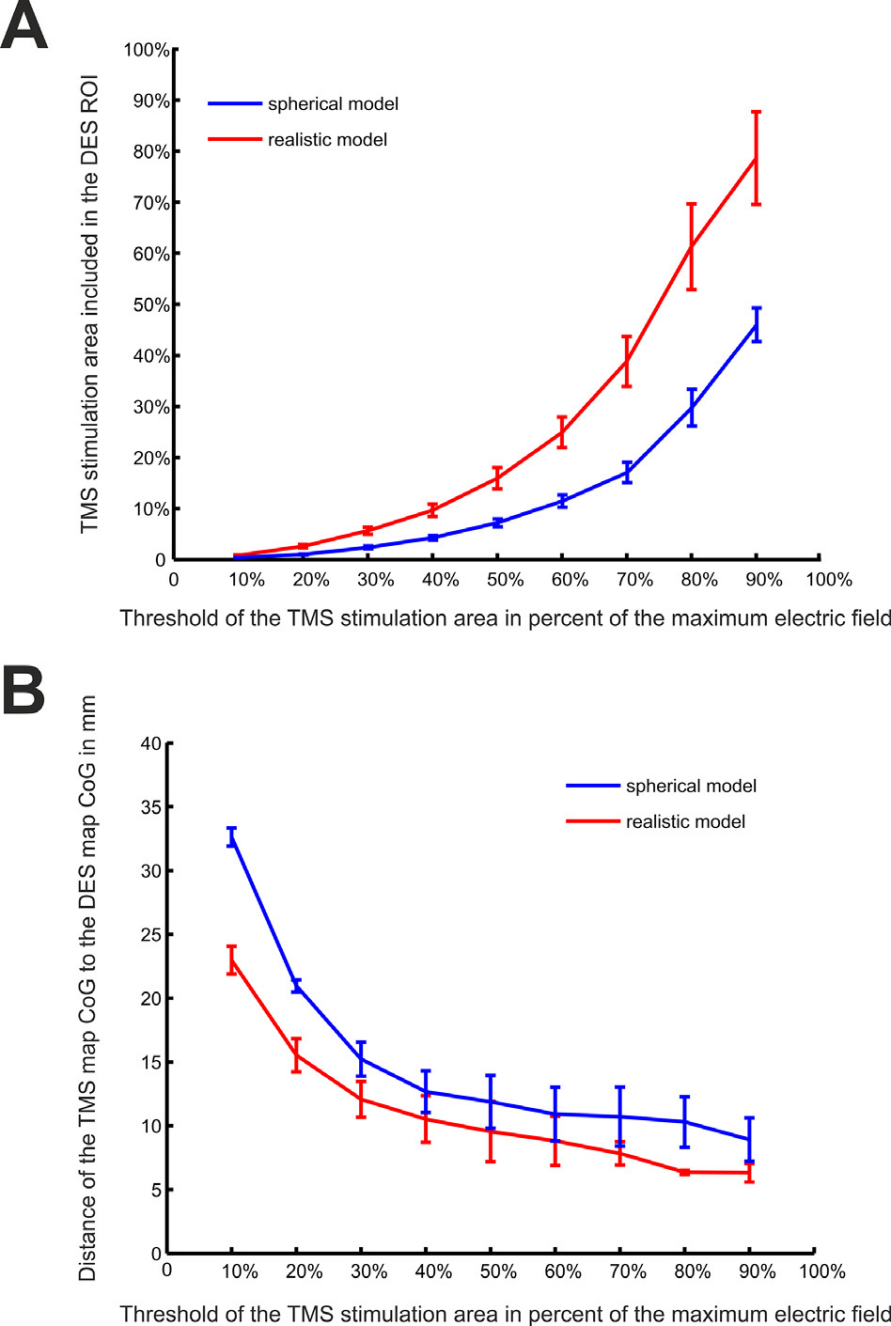
DES and TMS comparison: A) percentage of the overlap between the DES stimulation area (3.99 ± 0.46 cm^2^) and the stimulation area of the TMS for both the realistic model (red line) and the spherical model (blue line). Shown are mean ± standard error of mean over the six patients for the overlap between the DES and TMS stimulation areas for different thresholds of the TMS electric field. For increasing TMS electric field strengths an increasing percentage overlaps with the DES ROI. This effect is more pronounced for the realistic model than for the spherical model. B) Distance between the CoG of the TMS map and the CoG of the DES map for both the realistic and the spherical model. Shown are mean ± standard error of mean over the six patients for the distance between the DES and TMS CoGs for different thresholds of the TMS electric field. With increasing TMS electric field threshold, the distance between the TMS and DES CoGs decreases.

To analyze the mapping accuracy of the TMS computational model we computed the percentage of the area of the *E_CoG_realistic_* in a DES determined region of interest (Fig. 4D for an example) for different electric field thresholds. By increasing the threshold, a higher percentage of the electric field was included in the DES ROI (Fig. 5A). For the realistic model about 80% of its highest *E_CoG_realistic_* (>90% of the maximum) area fell into the DES ROI. In comparison, for the spherical model a smaller percentage of its highest *E_CoG_sphere_* was included in the DES ROI. The differences between the realistic and the spherical model were mainly due to the larger stimulation area determined by the spherical model (see Table 1 for the covered area on the brain for both the interpolated spherical and the realistic model). The better overlap of the realistic compared to the spherical model was independent of the threshold chosen to determine the DES ROI (Supplementary Fig. 1). A similar trend as for the stimulation area overlap between DES and TMS was found for the Euclidian distances. With increasing electric field threshold, the Euclidian distance for the TMS map CoG to the DES map CoG decreased (Fig. 5B). For the 90% threshold the distance was 6.3 ± 0.7 mm for the realistic and 8.9 ± 1.7 mm for the spherical model. For the model free simple estimate of the DES CoG the Euclidian distances to the TMS map CoGs were 9.4 ± 1.5 mm for the realistic and 11.0 ± 1.5 mm for the spherical model for the 90% threshold, respectively.

## 5 Discussion

In this study we investigated the mapping accuracy of transcranial magnetic stimulation for determining motor areas by comparing it to the gold standard of direct electrical stimulation. We found that the TMS stimulation area determined by a computational approach significantly overlaps with the computed DES stimulation area (see Fig. 4, Fig. 5). Especially in the area of highest electrical field strength, the overlap was strongest. Also the distance between the TMS map CoG and the DES map CoG was shortest for the highest electric field strengths. Using a simplified approach which does not depend on a computational model to estimate the DES CoG results in slightly larger distances, which indicates that a merely point like stimulation approximation in DES might not be appropriate but nevertheless can give another validation independent of the TMS computational model.

Compared to spherical models, realistic models make a more specific prediction of TMS target areas which are in better accordance with the DES results. Spherical models are not able to account for effects caused by the gyrification of the brain. The secondary electric field caused by charge accumulation at the tissue interface between CSF and GM can have a profound influence on the electric field distribution mainly leading to enhanced electric fields at the gyral crowns perpendicular to the coil orientation (Thielscher et al., 2011). Thus, taking into account the gyrification of the brain surface causes a spatially more specific prediction than that of a spherical model. The superior overlap of the realistic model compared to the spherical model with the DES stimulation area indicates that for a precise estimation of TMS target areas an accurate representation of the cortical foldings seems necessary. The good overlap of the TMS stimulation area of the realistic model with the DES gold standard also validates the use of individual FEM models as being able to reliably predict brain regions excited by TMS. While in this study we focused on the motor cortex, potentially TMS FEM simulations might also help in predicting stimulation sites that are non-motor related, for example brain regions that are related to speech.

While it is difficult to put an exact number to the spatial extent of the excitation area of DES and TMS, a few estimates can be made. The radius of the current spread of direct electrical stimulation can be estimated by the formula: *I* = *Kr*^2^ (Ranck, 1975; Stoney et al., 1968), with *I* the current strength in µA, *K* the current-distance constant which was estimated to be *K* = 1292 µA/mm^2^ by Stoney et al. (1968) and *r* the radius of the stimulation area in mm. Based on this formula, the stimulation radius can be estimated to lie between 2 and 4 mm for current strengths between 5 and 20 mA as applied in this study. This might lie in a similar range as has been estimated for deep brain stimulation (DBS) (McIntyre et al., 2004). However, the effective stimulation area of DES might be a factor of two or three larger than estimated as was shown by combining microstimulation with fMRI (Tolias et al., 2005). Despite this, the stimulation area of DES seems to be in the range of a few mm^2^ while the predicted stimulation area of TMS spans several cm^2^ which can extend over one or two neighboring gyri. Thus, a millimeter precise stimulation of cortical tissue in a noninvasive manner does not seem possible with currently employed TMS coils. Other noninvasive brain stimulation methods like transcranial focused ultrasound (Legon et al., 2014; Tufail et al., 2010) stimulation might be able to overcome the physical constraints of transcranial magnetic stimulation.

One possible limitation of this study is that the area that was directly stimulated during surgery was limited in its spatial extent. As only as much skull and dura was removed as was necessary to have access to the tumor, the brain area that was accessible with DES was limited. Thus, the estimated DES stimulation area might be larger than that estimated by the applied procedure. However, reference points that were recorded further apart from the motor areas did not elicit any MEPs even with highest stimulation amplitudes. Also, in every patient there were DES stimulation points that did not result in MEPs at all, thus demonstrating that there was a spatial confinement in the measurement data. Another point we cannot address in this study is the possible influence of neuronal elements deeper in the sulcal wall as they were not accessible with the applied preparation during surgery. Also we did not take conductivity anisotropy into account which exerts stronger effects in deeper WM regions but is likely to be negligible in superficial GM (Opitz et al., 2011). Thus, we deliberately focused on comparing the surface effects of TMS and DES. The influence of the tumor on the electric field distribution resulted in nonnegligible changes only in the case of very high tumor conductivities, which likely did not occur in our study. Although it seems unlikely that our results are significantly dependent on tumor conductivity, future studies might profit from a direct conductivity measure of the tumor using Magnetic Resonance Impedance Tomography (Minhas et al., 2011). The mapping accuracy of TMS can be possibly improved by employing more than one TMS coil orientation which was not performed in this study to keep the experimental time for the patients as short as possible. Locally enhanced mesh resolution around the stimulation electrode can improve the numerical precision of the DES results in future studies. Finally, mapping accuracy depends on the precision of the neuronavigation procedure which is estimated to have an uncertainty of ca. 5 mm (Ruohonen and Karhu, 2010).

Future studies can possibly improve on predicting the stimulation area by taking into account other factors like the orientation and morphology of the targeted neuronal elements (Radman et al., 2009) which determine their degree of excitability by external electric fields as was already addressed in Salvador et al. (2011) or Pashut et al. (2011). However, in the absence of precise knowledge of these factors the absolute electric field strength seems to be a robust measure to estimate stimulation areas. In this study we focused on the direct stimulation effects of the electric field, however it is generally assumed that TMS acts on brain circuits (Dayan et al., 2013; Fox et al., 2012) and exerts its effect on remote brain areas as well. In conclusion, our results suggest that TMS combined with computational electric field modeling can reliably predict stimulation areas that show large overlap with computationally predicted DES areas. Even though the more commonly employed spherical models do not miss the stimulation area, their spatial specificity is limited compared to more realistic models. This computational approach can possibly be used to more precisely determine eloquent motor areas in a noninvasive manner before brain surgery. It has to be tested in future studies if this method can help to improve clinical outcomes for patients undergoing brain tumor resection.

## Appendix Supplementary materials

Supplementary for validating computationally predicted TMS stimulation areas using direct electrical stimulation in patients with brain tumors near precentral regions.

## Footnotes

Supplementary material associated with this article can be found, in the online version, at doi:10.1016/j.nicl.2014.03.004.
