# Supplementary material for: Validating computationally predicted TMS stimulation areas using direct electrical stimulation in patients with brain tumors near precentral regions
Source: Neuroimage Clin. 2014 Mar 18;4:500–7. doi: 10.1016/j.nicl.2014.03.004 (PMC3984442; doi:10.1016/j.nicl.2014.03.004)
Supplement: Supplementary file 1 — Supplementary for validating computationally predicted TMS stimulation areas using direct electrical stimulation in patients with brain tumors near precentral regions. [file mmc1.docx]

**Supplementary material**

*Influence of the DES ROI*

*Methods*

To study the influence of the DES ROI on the comparison between the TMS stimulation area and the DES stimulation area we systematically varied the electric field threshold to determine the DES ROI from 10% to 90% of the maximum in steps of 10%. The chosen threshold determines the spatial extent of the ROI from a more extended area to a more confined area with increasing threshold. The overlap between the DES and TMS area was then computed for each DES ROI separately. The same procedure was performed for the distance between the TMS map CoGs and the DES CoGs.

*Results*

Irrespective of the chosen DES ROI, the realistic model showed higher overlap with the DES area than the spherical model (see Fig S1). Also the distance of the CoG for the realistic model was shorter than the distance of the CoG of the spherical model irrespective of the chosen DES ROI (data not shown).

*Influence of the tumor conductivity.*

*Methods*

We studied the influence of the tumor conductivity on the electric field distribution in one subject in more detail. The tumor was located in the postcentral gyrus (see Fig. S2A left panel). To study the maximal effect that might occur for an altered tumor conductivity, we placed the TMS coil directly above the tumor region in the simulation. We then systematically varied the tumor conductivity from 0.01 S/m up to 1.5 S/m for ten different conductivity values while the other tissue conductivities were kept constant and calculated the TMS induced electric field for each configuration. Subsequently, we calculated the relative difference in the electric field (in the area above 50% of the maximum of the reference electric field) with respect to the tumor conductivity which we employed in the study$(\sigma_{tumor}$ = $\sigma_{WM}$) on the GM surface.

*Results*

The reference electric field distribution $(\sigma_{tumor}$ = $\sigma_{WM})$to which we compared the other simulation results is shown in Fig. S2A (right panel). For low tumor conductivities changes in the electric field were small with maximal changes of 5% (data not shown). Also for strongly enhanced tumor conductivities $\sigma_{tumor}$ = 0.5 S/m changes in the electric field were moderate (Fig. S2B left panel). Only for tumor conductivity values already in the range of CSF $\sigma_{tumor}$ = 1.5 S/m, stronger changes showing a decrease in the electric field of ca. 25% (Fig. S2B right panel) are present. In this case the high tumor conductivity drags current to deeper regions leading to a reduction at the cortical surface.


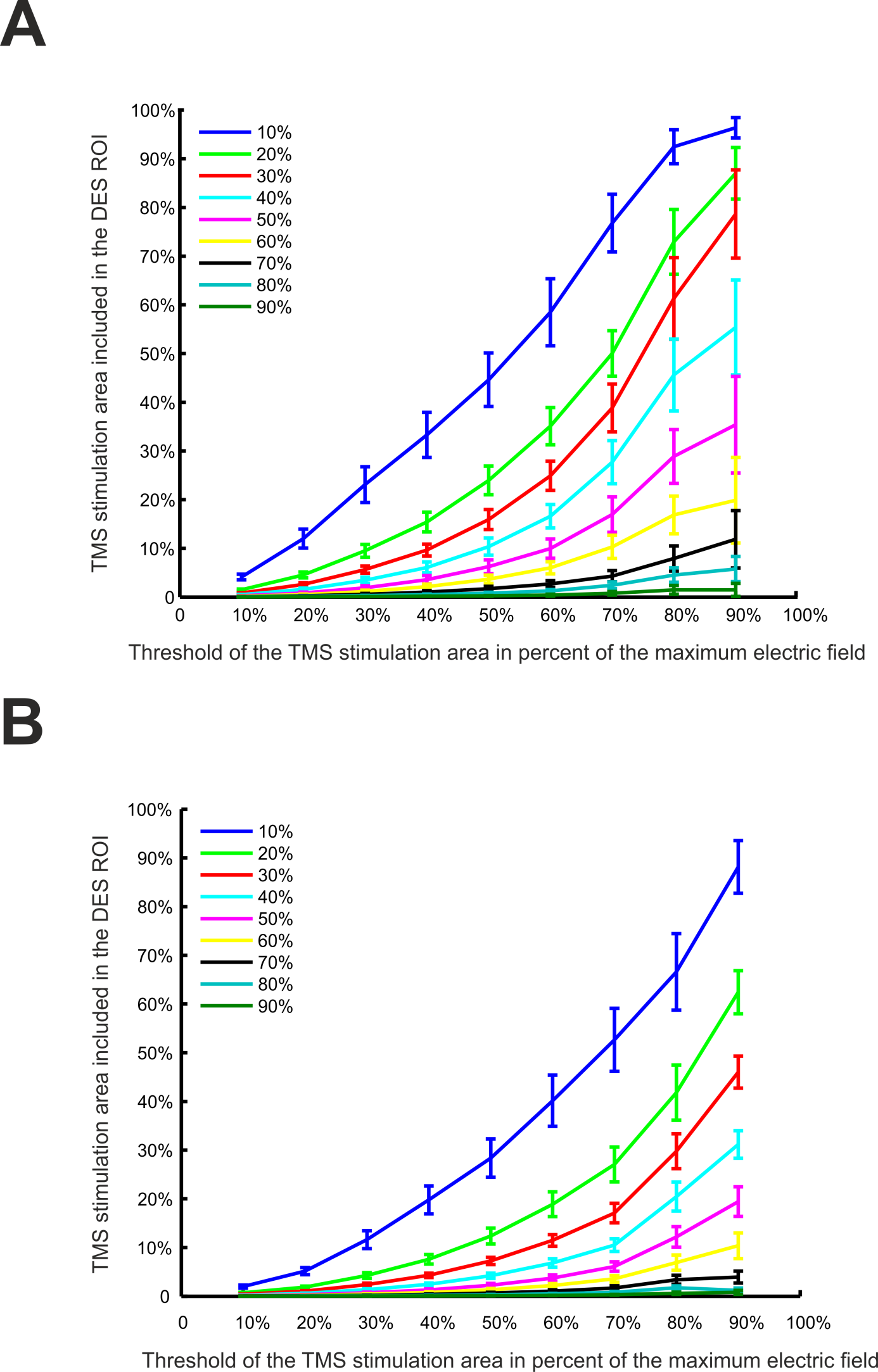


Supplementary Figure 1: DES and TMS overlap for different thresholds of the DES ROI for the realistic model **A)** and the spherical model **B)**. Shown are the overlaps for different ROIs (based on different electric field thresholds, from 10% to 90% of the maximum, different colored graphs) of the DES stimulation area. For smaller thresholds the DES stimulation area is increased and with it also the overlap with the TMS electric field. Irrespective of the exact threshold the graphs show all the same tendency that with increasing TMS field strength a higher percentage of the TMS electric field is included in the DES ROI.


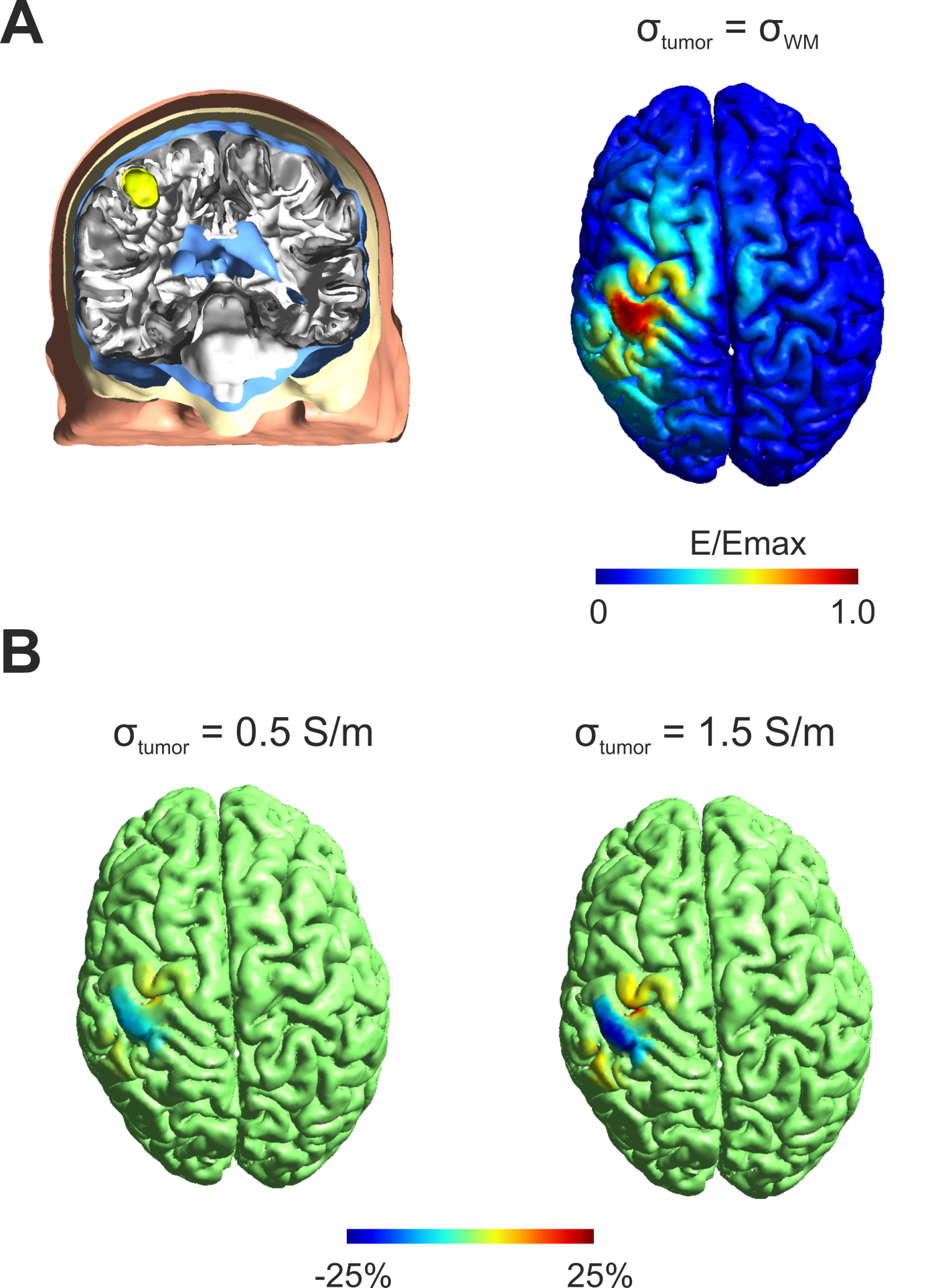


Supplementary Figure 2: Influence of the tumor conductivity on the TMS electric field distribution. **A)** Coronal cut-through image of the head model showing the extent of the tumor. The tumor was located in the postcentral gyrus (left panel, tumor in yellow). The TMS electric field when placing the coil directly above the tumor location in the postcentral gyrus is shown for $\sigma_{tumor}$ = $\sigma_{WM}$ (right panel). Highest electric fields occur at the top of the postcentral gyrus. **B)** Difference in the electric field between the $\sigma_{tumor}$ = $\sigma_{WM}$ and $\sigma_{tumor}$ = 0.5 S/m (left panel) and $\sigma_{tumor}$ = 1.5 S/m (right panel) simulations. Only the assignment of very high tumor conductivities leads to a local decrease in the electric field of up to 25% in the brain regions above the tumor.
